# FM-Adapt: Foundation model adaptation with photoacoustic-supervised learning for interventional ultrasound^☆^

**DOI:** 10.1016/j.pacs.2026.100822

**Published:** 2026-03-21

**Authors:** Jahid Hasan, Praveenbalaji Rajendran, Ying Cai, Manojit Pramanik

**Affiliations:** a Department of Computer Science, Iowa State University, Ames, IA, USA; b Department of Radiation Oncology, Winship Cancer Institute, Emory University, Atlanta, GA, USA; c Department of Electrical and Computer Engineering, Iowa State University, Ames, IA, USA

**Keywords:** Foundation model, Photoacoustic imaging, Ultrasound imaging, Interventional guidance, Segmentation

## Abstract

Foundation models (FMs), such as the Segment Anything Model (SAM), have remarkable capabilities for general-purpose segmentation tasks through large-scale pre-training. However, a substantial domain shift limits their effectiveness in complex medical imaging. Here we introduce FM-Adapt, the first parameter-efficient adaptation of a FM (SAM-based vision transformer) into a resolution-agnostic architecture with photoacoustic (PA)-supervised learning for dual-target interventional ultrasound (US) segmentation. We demonstrate FM-Adapt in the context of PA-supervised interventions, specifically for US-guided needle tracking and simultaneous target identification (breast tumor segmentation). We train once with this unified adaptation framework to produce two specialized model weights: USPA-SAM for real-time tracking of needles and BT-SAM for segmenting breast tumors. This framework utilizes frozen pre-trained encoder components and fine-tunes only the mask decoder, allowing the model to process native (256 × 256) clinical images without spatial degradation while achieving state-of-the-art performance with high computational efficiency. USPA-SAM achieves a mean modified Hausdorff Distance (MHD) of 0.34 mm, a targeting error (TE) of 0.83 mm, and a 100% needle localization success rate (NLSR), outperforming baselines by a factor of 3–17× in spatial precision. Notably, on tumor segmentation, BT-SAM achieves Dice scores of 93.6% and 96.3%, along with IoU scores of 89.2% and 94.0%, demonstrating strong generalization to unseen data. This work demonstrates that our models achieve a 27× improvement in computational efficiency to process native clinical images at 34 FPS on a single GPU to enable real-time clinical adaptation.

## 1 Introduction

Photoacoustic imaging (PAI) is a hybrid noninvasive imaging modality that combines both optical and ultrasound (US) imaging [1], [2], [3], [4], [5], [6], [7]. It has the potential to be used in clinical settings to leverage well established clinical US imaging systems for surgical and interventional guidance [8], [9], [10]. It supports various diagnostic and interventional treatments, including cancer classification, breast imaging, soft-tissue inspection, and the guidance of biopsies for the kidney, brain, and lymph node [11], [12], [13], [14], [15]. In interventional guidance, photoacoustic (PA) imaging provides critical contrast [16] to identify specific target pathology (*e.g.,* tumor), whereas US helps with real-time instrument navigation (*e.g.,* needle tracking) to create a seamless guidance workflow [8], [10], [17]. However, the clinical translation of photoacoustic imaging system remains challenging due to various factors [18]. As an intermediate step toward clinical application, PA-supervised learning can enhance interventional ultrasound by providing high-quality training data for needle tracking tasks [19], [20]. In this approach, PA images of the needle are used as ground truth to train deep learning (DL) models, thereby improving the US-guided needle tracking procedure. This approach eliminates the need for time-consuming, expert-led manual segmentation to obtain the ground truth required for DL model training. As Conventional US-based image processing methods for needle tracking application use expert-led manual segmentation to generate training data and are designed for task-specific single target segmentation. Such architectural complexity presents significant challenges to adapting new target data for interventional guidance. As a result, dual-target segmentation to track both needle and tumor regions during an interventional ultrasound procedure remains an open problem.

In recent years, artificial intelligence (AI), particularly deep learning (DL) [21], [22], [23], [24], [25] such as U-Net [26], UIU-Net [19], ForFormer-UNet [20], ASCENT+ [24], and DeepLab [27], has become an integral part to improve decision-making processes in medical imaging such as tissue segmentation, lesion detection, and needle localization. However, existing DL-based approaches are designed for task-specific, single-segmentation to identify distinct anatomical lesion features and outline tissue boundaries. For US-guided interventional needle tracking, not only we need to improve the needle visibility, but we also need to segment the target region (tumor, target organ, etc.). This will help the ultrasound operators/clinicians to visualize the needle better and also identify the structure where the needle needs to go. During real clinical procedures, clinicians expect to simultaneously track the needle (a thin, linear, and highly echogenic bright line) and delineate the target pathology (an irregular, heterogeneous morphology with ill-defined boundaries, as seen in the presenting tumor), without requiring manual supervision. This requires dual-target segmentation. Although, earlier approaches [19], [20], [24] proposed needle segmentation using PA ground truth images, but this would require a separate task-specific workflow (another DL network) for tumor (or any other target) segmentation. Additionally, these DL models demand high computational resources to develop a task-specific training pipeline, extensive labeled data, and manual hyperparameter tuning. Such complexity of design makes them impractical to use for clinical settings, particularly for dual-target (or multi-target) segmentation problems, where such task-specific architectures introduce significant computational burden for clinical deployment. Overall, these issues underscore the need for an adaptive, computationally efficient solution that can perform both tasks in real time to track the needle and segment the tumor without relying on extensive manual annotation, thereby enhancing procedural workflow and precision.

Recently, foundation models (FMs) [28], [29] in computer vision have demonstrated the potential for general-purpose visual understanding on a large-scale supervised and unsupervised pre-training. Building FMs is very resource-intensive, requiring sophisticated infrastructure, long training hours, and advanced hardware (GPUs). In contrast, adapting an existing FM for a specific task is far less costly, as it leverages pre-trained capabilities and typically requires only fine-tuning on smaller, task-specific datasets. Unlike traditional task-specific DL models, these general-purpose FMs, such as Segment Anything (SAM) [30], show significant capabilities but fall short on medical imaging due to the substantial domain shift between natural and medical images [31]. To address this domain shift, most efforts to adapt SAM for medical imaging use a pre-training and fine-tuning approach [32] to utilize unlabeled data in a self-supervised manner. To adapt SAM for medical imaging [33] tasks, several adaptation approaches have been proposed such as MedSAM [34], SAMUS [35], [36], CEUS-SAM [37], and KnowSAM [38], Masked Autoencoders (MAEs) [39], MaskFormer [40]. These adaptations demonstrate the potential to utilize pre-trained FMs without requiring extensive re-training or task-specific prompting. At the same time, such vision FMs are trained on massive datasets and learn general-purpose visual representations. This enables these models to adapt efficiently to various downstream tasks [28] with minimal labeled data. This inherent multi-task learning capability of FMs enables us to address complex PA-supervised interventions and solve the dual-target US segmentation challenge, which has not been addressed in the literature.

To address the problems in PA-supervised US interventions, we introduce FM-Adapt, the first framework for adapting FMs to real-time dual-target segmentation. The proposed approach solves two key challenges simultaneously: (i) manual annotation bottleneck that requires multiple expert annotators to label the data for training, which are prone to bias, time-consuming and expensive, and (ii) unifying needle tracking and tumor segmentation within a single, parameter-efficient architecture that generalizes across different US imaging conditions and anatomical features. Unlike traditional deep learning models, which require separate networks for each task, FM-Adapt uses a standardized adaptation protocol by freezing the SAM encoder components and fine-tuning only the mask decoder to produce two task-specific specialized models. This standardized adaptation protocol generates: USPA-SAM (combining ultrasound and photoacoustic) for PA-supervised US-guided needle tracking and BT-SAM for **b**reast **t**umor segmentation. FM-Adapt is the first framework to demonstrate parameter-efficient adaptation of FMs in PAI. Our method utilizes a pre-trained SAM-based vision transformer (ViT) model for dual-target segmentation, where we efficiently fine-tune only the mask decoder (4M parameters) while freezing the image and prompt encoders to reduce computational resources and training time. This design approach enables us to be resolution-agnostic in processing our native 256 × 256 clinical data without degradation in resolution. Our approach comprehensively learns multi-class target regions and effectively adapts to the lesions and anatomical structures of US images within a unified architecture. The comprehensive evaluation results demonstrate that our standardized adaptation protocol with dual specialization enables effective transfer learning to handle cross-domain generalization of US-PA images robustly in varied settings. Our main contributions are:

• We introduce FM-Adapt, the first parameter-efficient framework that adapts a FM for dual-target segmentation in US imaging for PA-supervised interventions, producing two specialized models (USPA-SAM and BT-SAM) through a standardized adaptation protocol that needs no task-specific architectural modifications.
• We demonstrate that a unified adaptation framework with dual specialization effectively generalizes across distinct segmentation tasks in real-time needle tracking and tumor segmentation.
• Our comprehensive evaluation on multiple datasets shows the proposed method outperforms state-of-the-art methods by improving the spatial accuracy of MHD by 0.34 mm and TE by 0.83 mm for needle localization, up to 3–17× on USPA-SAM. Meanwhile, BT-SAM achieves Dice scores of 93.6% and 96.3% on the benchmark dataset, respectively.

## 2 Methods

### 2.1 Architectural overview

**Fig. 1:**
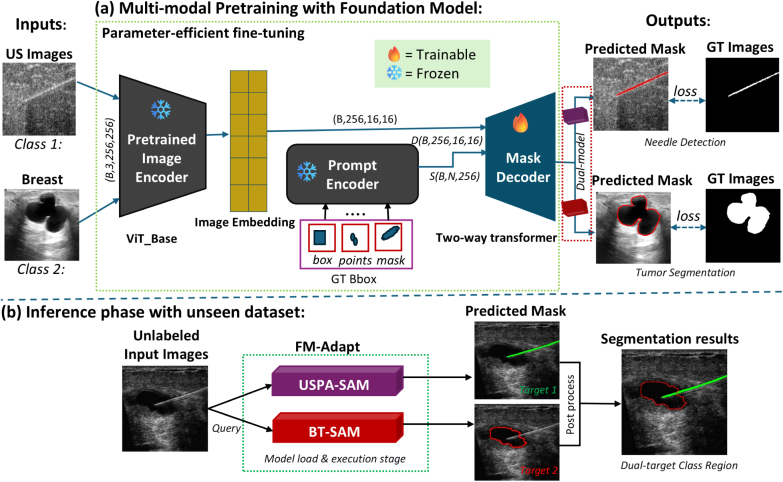
Overview of the FM-Adapt framework. (a) **Training Phase:** Parameter-efficient fine-tuning strategy for PA-supervised US interventions, the inputs are represented as two target classes (needle and breast tumor). The predicted needle and tumor masks are shown in red; (b) **Inference Phase:** The adapted FM-Adapt backbone produces two specialized models: USPA-SAM for needle tracking and BT-SAM for tumor segmentation. Predicted masks for *target 1:* US needle in green and *target 2:* US tumor in red. Final segmentation results (needle in green, tumor in red) are shown after post-processing.

The proposed FM-Adapt framework adapts the SAM [30] architecture as illustrated in Fig. 1. During training, the weights of the image and prompt encoders are frozen to reduce computational overhead and training time, while only fine-tuning the mask decoder (4M parameters) to learn class-specific needle and tumor features shown in Fig. 1(a). This approach significantly reduces the number of trainable parameters, making the framework feasible to train on a single GPU (NVIDIA A100, 40 GB VRAM). We train once with this same framework and architecture, where the decoder simultaneously learns both target classes to produce two specialized model weights: USPA-SAM for real-time needle tracking and BT-SAM for breast tumor segmentation. Both models enable simultaneous visualization of both targets during US-guided intervention by leveraging a frozen encoder backbone and a trainable mask decoder. Importantly, this whole process is performed with a single joint training run in parallel using the same mask decoder that learns distinct output tokens for each class, without requiring separate training runs or task-specific architectural modifications.

Fig. 1(b) shows the inference phase, where FM-Adapt utilizes unlabeled US images only (no PA input at inference) as input to query. It then employs an automatic prompting strategy based on a task-specific bounding box to eliminate the need for user input or ground truth. This generated prompt then guides each specialized model’s weights to produce class-specific predicted masks for both target classes (*Target class 1:* needle in green and *Target class 2:* tumor in red) with precision. These predicted masks are combined in a post-processing step to produce simultaneous dual-target segmentation results.

#### 2.1.1 Image encoder backbone

We utilized a pre-trained SAM-based vision transformer base model (ViT-B) as the image encoder. Initially, this model was trained on 1024 × 1024 images, where we adapted it to process native 256 × 256 clinical images without resizing and losing the original image resolution. To preserve resolution-agnostic, we replaced the absolute positional embeddings with relative positional encoding [41], and the input is divided into 16 × 16 non-overlapping patches (also known as a token grid) using bilinear interpolation. This adaptation guarantees that the spatial information preserves fine-grained details while providing better generalization of images for varying input grid sizes. Finally, the adapted ViT processes feature maps of dimensionality appropriate for our input images in the transformer, and then passes them to the mask decoder. Meanwhile, the weights of the image encoder remain frozen during model training.

**Fig. 2:**
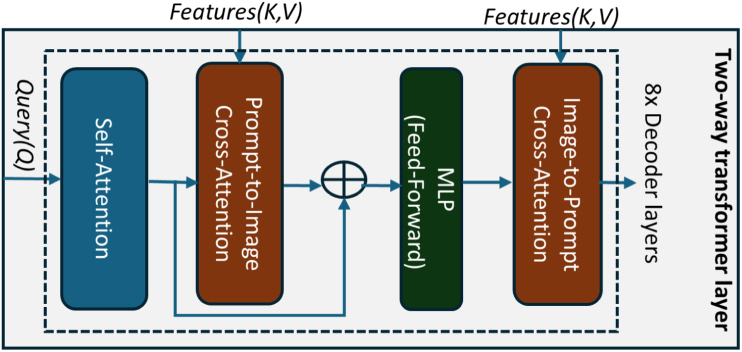
The schematic details of the two-way transformer mask decoder layer.

#### 2.1.2 Prompt encoder

The prompt encoder uses the ground truth (GT) bounding box to translate user input, such as boxes, points, and masks, into a unified 256-dimensional embedding. In our framework, we provide GT bounding boxes as a pair to learn the embedding via positional encoding, allowing us to coordinate at different positions. This process captures the spatial relationships to benefit the model in localizing thinner needle tips. This prompt-based approach enables the model to learn from both sparse and dense semantic embeddings, which serve as the input to the mask decoder. During model training, the prompt encoder remains frozen while adapting to US imaging features through the trainable mask decoder.

#### 2.1.3 Mask decoder and cross-attention block

The two-way transformer mask decoder illustrated in Fig. 2 is the only trainable component in our fine-tuning adaptation. It processes both image and prompt embeddings bidirectionally to enable the contextual information of the US-PA images, image features, and semantic prompts to adapt US image patterns. Where, let token $T\in R^{N_{t}\times D}$ represent the learnable query tokens concatenated with prompt embeddings and $I\in R^{H\times W\times D}$ be the frozen image embeddings from the pre-trained image encoder where D=256 is the feature dimension. Each transformer layer in our method has four sequential functions that enable bidirectional attention flow, enabling domain-specific adaptation of US imaging. For needle segmentation, it comprehends the thin, linear structures of the needle, including the angle of needle insertion, needle tip lengths, and sizes, while negating the consequences of acoustic shadows and speckle noise to infer the occluded needle structure. For tumor segmentation, it adapts to the irregular edges and heterogeneous texture patterns of breast lesions, allowing the same model architecture to enable robust generalization in dual-target class regions.

**Token Self-Attention:** In this multi-head attention mechanism, where tokens (T) act as queries (Q), image features as keys (K) and values (V): $T^{\prime }=\mathrm{LN}\left(T+\mathrm{SelfAttn}\left(Q=T,K=T,V=T\right)\right)$, where LN denotes Layer Normalization, and SelfAttn denotes Self-Attention.

**Prompt-to-Image Cross-Attention:** The tokens updated from the self-attention ($T^{\prime }$) acts as queries in cross-attention (CrossAttn) to select and extract the most relevant image features from the image embedding (I): $T^{\prime \prime }=\mathrm{LN}\left(T^{\prime }+\mathrm{CrossAttn}\left(Q=T^{\prime },K=I,V=I\right)\right)$. Here, tokens act as queries Q that selectively attend to spatial positions in the image embeddings (keys K and values V) to help identify which regions is most relevant to these semantic prompts.

**MLP Transformation:** The updated token embeddings ($T^{\prime \prime }$) are processed by a point-wise multi-layer perceptron (MLP) block as a feed-forward network (FFN) with a GELU activation function is used to the token embedding: $T^{\prime \prime \prime }=\mathrm{LN}\left(T^{\prime \prime }+\mathrm{MLP}\left(T^{\prime \prime }\right)\right)$, where $\text{MLP}\left(x\right)=W_{2}\cdot \text{GELU}\left(W_{1}x+b_{1}\right)+b_{2}$, with an intermediate hidden dimension. This introduces non-linearity, enabling models to learn the complex patterns and domain-specific feature combinations.

**Image-to-Prompt Cross-Attention:** In the final stage, we applied a reverse attention flow to update the image feature understanding based on the refined prompt context. This process enables the distillation of image features based on prompt details, allowing for the analysis and contextualization of precise image regions. This is formulated as: $I^{\prime }=\mathrm{LN}\left(I+\mathrm{CrossAttn}\left(Q=I,K=T^{\prime \prime \prime },V=T^{\prime \prime \prime }\right)\right)$. This allows the image features to be distilled and contextualized by the prompt information.

Overall, this bidirectional attention mechanism constructs a feedback loop to guide the attention layer to anatomically pertinent structures (such as the needle and tumor), enabling context-aware feature representation and refined semantic understanding in the backward direction to improve segmentation accuracy for both target classes.

#### 2.1.4 Dual-target mask prediction

Our framework represents the first novel approach to handle dual-target segmentation within a single model architecture, efficiently balancing segmentation performance and computational overhead. This design decision aims to prevent task inference complexity for needle tracking and tumor segmentation, since each class requires learning distinct feature representations. For example, localizing a thin linear geometry for a needle depends on capturing high-contrast geometric features, whereas segmenting tumors demands sensitivity to amorphous structural patterns. Our decoder is designed to generate predictions for both targets into task-specific outputs simultaneously. After processing through 8 decoder layers, the updated image embeddings $I_{\text{final}}$ are upsampled by a factor of 4 using two transposed convolution layers. The final output tokens $T_{\text{final}}$ are passed through the MLP to produce a set of mask embeddings. The final mask logits $M_{\text{logits}}$ is generated using dot product as follows: $M_{\text{logits}}=\text{MLP}\left(T_{\text{final}}\right)\cdot I_{\text{upsampled}}$

#### 2.1.5 Loss functions

To effectively train the model for this dual-target complex segmentation task, we use a composite loss function $L_{\text{total}}$, which is the weighted sum of three distinct components: Binary Cross-Entropy (BCE), Dice, and Focal losses. The unified loss is formulated as a weighted sum:

(1) $$L_{\text{total}}=\underset{\text{pixel-wise supervision}}{\underset{︸}{w_{\text{bce}}L_{\text{bce}}}}+\underset{\text{segmentation overlap}}{\underset{︸}{w_{\text{dice}}L_{\text{dice}}}}+\underset{\text{class imbalance}}{\underset{︸}{w_{\text{focal}}L_{\text{focal}}}}$$

In our experimental evaluation, we found the weighting of $w_{\text{bce}}=0.3,w_{\text{dice}}=0.4,\text{and}w_{\text{focal}}=0.3$ provided better class balance for the model.

**Binary Cross-Entropy (BCE) Loss** provides pixel-wise supervision for classification tasks:

(2) $$L_{\mathrm{bce}}=-\frac{1}{N}\overset{N}{\underset{i=1}{\sum }}\left[y_{i}\log\left(\sigma \left({\overset{ˆ}{y}}_{i}\right)\right)+\left(1-y_{i}\right)\log\left(1-\sigma \left({\overset{ˆ}{y}}_{i}\right)\right)\right]$$

where $y_{i}\in \left\{0,1\right\}$ is the ground truth, ${\overset{ˆ}{y}}_{i}$ is the logit output of the model, σ(⋅) denotes the sigmoid function, and N is the total number of pixels.

**Dice Loss** optimizes segmentation overlapping between prediction and ground truth, which is necessary for thin needle structures to localize:

(3) $$L_{\mathrm{dice}}=1-\frac{2\overset{N}{\underset{i=1}{\sum }}\sigma \left({\overset{ˆ}{y}}_{i}\right)y_{i}+\epsilon }{\overset{N}{\underset{i=1}{\sum }}\sigma \left({\overset{ˆ}{y}}_{i}\right)+\overset{N}{\underset{i=1}{\sum }}y_{i}+\epsilon }$$

where $\epsilon =10^{-6}$ ensures numerical stability of the model.

**Focal Loss** handles severe class imbalance inherent in needle segmentation tasks:

(4) $$L_{\mathrm{focal}}=-\frac{1}{N}\overset{N}{\underset{i=1}{\sum }}\alpha {\left(1-p_{t}\right)}^{\gamma }\log\left(p_{t}\right),$$

$$\text{where}p_{t}=\left\{\begin{array}{cc} \sigma \left({\overset{ˆ}{y}}_{i}\right)\; & \text{if}y_{i}=1,\\ 1-\sigma \left({\overset{ˆ}{y}}_{i}\right)\; & \text{otherwise}. \end{array}\right)$$

where the factors α=0.8 and γ=2.0 are used for reducing the class imbalance and challenging misclassified pixels.

All target regions are normalized to a shape of [B,1,H,W] and match the original image size with the resized post-processing mask to align with our models 256 × 256 output resolution. Additionally, we also used label smoothing regularization (β=0.05), defined as $y_{i}^{\mathrm{smooth}}=y_{i}\left(1-2\beta \right)+\beta$, to reduce the model overfitting to the noisy and hazy structure of the US needle images.

### 2.2 Imaging data for training and testing

For needle segmentation, the training and testing experimental data were acquired using a dual-mode US+PA imaging system [42], [43], [44]. A Nd:YAG laser (Continuum, Surelite EX) [19] was used for PA imaging with 5 ns pulses at 10 Hz pulse repetition rate and 1064 nm wavelength illumination. For dual-mode image acquisition, a clinical US imaging system (ECUBE 12R, Alpinion, South Korea) [45], [46] was used in research mode to acquire simultaneous US+PA images. For experimental data collection, needles (18G, 23G BD PrecisionGlide, 1.2 mm by 38 mm; Franklin Lakes, NJ, USA) were inserted into *ex vivo* chicken tissue in a water tank, with ultrasound gel used for acoustic coupling. The needles were inserted parallel to the US transducers long axis at different depth levels and rotation angles. From this system, we collected approximately 2275 paired *ex vivo* US-PA images for training and validating our FM for needle tracking. This dataset was split into training (80%), validation (10%), and a held-out (10%) *ex vivo* test set to evaluate model performance.

To assess clinical generalization for needle tracking, we used *in vivo* human clinical US images from an open repository [47]. This acquisition of needle insertion utilizes a Fujifilm ultrasound imaging system [48] that contains clips and frames from real-time needle insertions, including breast and axilla procedures [47].

For the tumor segmentation task, we utilized the BUSI dataset [49], which comprises 780 breast ultrasound images from 600 female patients. The images were acquired using a LOGIQ E9 agile ultrasound system (GE Healthcare) with an ML6-15-D matrix linear probe and 1 MHz transducers. For robustness and evaluation purposes, we also utilized the BUS-UCLM lesion image dataset [50], which was collected using a Siemens ACUSON S2000TM ultrasound system and an 18L6 HD probe. A total of 683 images were acquired.

### 2.3 Metrics

We evaluated the performance of our FM using a comprehensive set of metrics for needle and tumor segmentation. For tumor segmentation, we evaluated Dice score, IoU, precision, recall, pixel accuracy, while for the complex needle tracking task we used MHD, TE, NLSR, and NLR metrics that is relevant for clinical applications.

#### 2.3.1 Intersection over union (IoU)

In this metric analysis, we used IoU, or Jaccard index [51] to assess the spatial segmentation overlap between a predicted segmentation mask $M_{p}$ and its corresponding ground truth mask $M_{g}$. These metrics are shown as follows:

(5) $$\text{IoU}=\frac{1}{N}\overset{N}{\underset{n=1}{\sum }}\frac{\left|M_{p}^{\left(n\right)}\cap M_{g}^{\left(n\right)}\right|}{\left|M_{p}^{\left(n\right)}\right|+\left|M_{g}^{\left(n\right)}\right|-\left|M_{p}^{\left(n\right)}\cap M_{g}^{\left(n\right)}\right|}$$

where IoU ranges from 0 (no overlapping) to 1 (perfect congruence).

#### 2.3.2 Dice coefficient

The Dice coefficient (or Dice Similarity Coefficient DSC or F1 score) [52] estimates the overlap region divided by the total number of pixels in both segmentation masks:

(6) $$\text{DSC}=\frac{1}{N}\overset{N}{\underset{n=1}{\sum }}\frac{2\cdot \left|M_{p}^{\left(n\right)}\cap M_{g}^{\left(n\right)}\right|}{\left|M_{p}^{\left(n\right)}\right|+\left|M_{g}^{\left(n\right)}\right|}$$

The bounded range from 0 to 1 allows for precise detection of the boundary in image segmentation, as DSC is usually more susceptible to true positive metrics used in medical image segmentation.

#### 2.3.3 Precision

This metric is used to evaluate the accuracy of the positive predictions by calculating the fraction of all predicted pixels (True Positives) relative to the total number of pixels predicted as positive in the segmented region [53]. The computed average across all N samples is:

(7) $$\text{Precision}=\frac{1}{N}\overset{N}{\underset{n=1}{\sum }}\frac{\left|M_{g}^{\left(n\right)}\cap M_{p}^{\left(n\right)}\right|}{\left|M_{p}^{\left(n\right)}\right|}$$

where, $M_{g}^{\left(n\right)}$ and $M_{p}^{\left(n\right)}$ describing the ground truth positives (True Positives + False Negatives) and total predicted positive pixels (True Positives + False Positives) binary segmentation masks for the n-the sample, respectively. This measurement helps in minimizing false positives.

#### 2.3.4 Recall

The recall or sensitivity is a quantitative measure that assesses the proportion of the actual positive pixels in the target structure [48] that are correctly identified by the model. It is defined as:

(8) $$\text{Recall}=\frac{1}{N}\overset{N}{\underset{n=1}{\sum }}\frac{\left|M_{g}^{\left(n\right)}\cap M_{p}^{\left(n\right)}\right|}{\left|M_{g}^{\left(n\right)}\right|}$$

where, $M_{g}^{\left(n\right)}$ is the total ground truth positives (True Positives + False Negatives) pixels.

#### 2.3.5 Pixel accuracy

This pixel accuracy metric helps to measure the accurate classified pixels of the entire image, and for a multi-class segmentation task with K classes can be defined as:

(9) $$\text{Pixel Accuracy}=\frac{\overset{K}{\underset{k=1}{\sum }}{\mathrm{TP}}_{k}}{\overset{K}{\underset{i=1}{\sum }}\overset{K}{\underset{j=1}{\sum }}C_{ij}}$$

where, $C_{ij}$ means the total number of pixels of true class i predicted as class j, and k is the sum of all correctly classified true positives (TP) pixels.

#### 2.3.6 Modified hausdorff distance (MHD)

We used a Modified Hausdorff Distance (MHD), which extended the standard Hausdorff Distance that measures the sensitivity to outliers between predicted and actual needle boundaries. In the context of needle tracking and robustness to outliers, it quantified the maximum distance from any point in the predicted needle contour to the closest ground truth point. That provided an enhanced object matching of the worst-case mismatch between the predicted and ground truth needle segmentation. Consider two sets of points, $X_{p}=\left\{x_{1},x_{2},\ldots ,x_{{\text{Num}}_{X}}\right\}$ and $Y_{g}=\left\{y_{1},y_{2},\ldots ,y_{{\text{Num}}_{Y}}\right\}$, where $d\left(x,Y_{g}\right)=\min_{y\in Y_{g}}{\left\|x-y\right\|}_{2}$ denotes the minimum Euclidean distance from point x to the nearest point in set $Y_{g}$. The MHD is computed as the maximum of the above two average distances, ensuring that a symmetric and outlier-resistant measure as follows [52]:

(10) $$\text{MHD}=\max\left(\frac{1}{\left|X_{p}\right|}\underset{x\in X_{p}}{\sum }d\left(x,Y_{g}\right),\;\frac{1}{\left|Y_{g}\right|}\underset{y\in Y_{g}}{\sum }d\left(y,X_{p}\right)\right)$$

To maintain consistency with the experimental image resolution, we measured all MHD distances in pixel units and then converted to physical distance (mm).

#### 2.3.7 Targeting error (TE)

A clinically relevant metric for evaluating the precision of the target is the Targeting Error (TE), which was computed and quantified by the 2D Euclidean distance [19] between the minimum distances between the ground truth and the estimated needle lines to the center of the image. This metric measured the spatial accuracy of the predicted needle overlay. Let $d_{\text{abs}}$ represent the absolute distance between the image center and the actual needle line, and $d_{\text{seg}}$ represent the estimated distance between the two. The targeting error is then $\text{TE}=\left|d_{\text{abs}}-d_{\text{seg}}\right|$, which quantifies the spatial accuracy of the predicted needle overlay.

#### 2.3.8 Needle localization success rate (NLSR)

The NLSR computed the percentage of successful localization to gauge the algorithm’s ability to accurately locate the needle segmentation and further quantify its success rate [48]. Let $S_{g}$ be the ground truth needle pixel set, and $S_{p}$ be the set of predicted needle pixels. Two conditions must be satisfied for successful needle localization: (i) the estimated localization path should intersect the ground truth needle regions, and (ii) the overlap between the ground truth needle pixels $S_{gt}$ and the set of predicted needle pixels $S_{p}$ must exceed a minimum threshold: $\left|S_{g}\cap S_{p}\right|>0.5\times \min\left(\left|S_{g}\right|,\left|S_{p}\right|\right)$ [54]. This metric ensured both spatial accuracy and sufficient pixel-wise correlation between prediction and ground truth.

#### 2.3.9 Needle length ratio (NLR)

In relation to the actual length, the Needle Length Ratio (NLR) calculates how accurate the predicted needle length was [19]. Assume that the actual needle length $\ell_{\text{true}}$ and the predicted needle length $\ell_{\text{pred}}$, NLR is defined as $\text{NLR}=\ell_{\text{pred}}/\ell_{\text{true}}$. A ratio close to 1 indicated high precision in the length prediction to avoid the skewed results due to incorrect needle localization predictions by NLR.

## 3 Experimental analysis

### 3.1 Implementation details

We trained our framework on a high-performance computing (HPC) Nova cluster of Iowa State University (ISU) using a single NVIDIA A100 GPU with 40 GB of VRAM, CUDA 13.0, and PyTorch 2.9. We used the ViT-B model as the backbone with a batch size of 2 and image dimensions of 3 × 256 × 256 (channels × height × width). We fine-tuned the model for 30 epochs using the AdamW optimizer at a learning rate of $1\times 10^{-4}$. To ensure training stability, we used WarmUpCosineScheduler as it linearly increases the learning rate following a cosine annealing schedule, which helps the model converge effectively while avoiding early-stage model instability. We also applied gradient clipping with an L2 norm of 0.5 to ensure training stability and prevent exploding gradients in our vision transformer. During a single training phase, we compute gradients and update parameters only for the mask decoder (4M parameters) using weights from the pre-trained ViT-B (original pre-trained SAM-Base decoder weights) model, while the encoder components are kept frozen. This parameter-efficient approach significantly reduces GPU memory consumption, allowing the model to train for approximately 30 min on a single A100 GPU.

**Table 1:** Ablation study of three different ViT models as backbones.

Model	Emb. Dim	Depth	Heads	Size	Tunable params	GFLOPS	FPS
**ViT-B (Ours)**	256	2	8	93.7M	4M	32B	34
ViT-L	256	2	8	0.3B	4M	122B	16
ViT-H	256	2	8	0.6B	4M	259B	10

### 3.2 Ablation studies

We conducted a thorough ablation study using different vision transformer (ViT) model settings (ViT-Base as ViT-B, ViT-Large as ViT-L, ViT-Huge as ViT-H) to compare with our baseline pre-trained ViT-B model presented in Table 1 to determine the optimal balance of the model scaling and computation efficiency for deployment inclusivity rather than other methodological dimensions. All the models use the same mask decoder architecture containing an embedding dimension of 256, a depth of 2 transformer blocks, and 8 attention heads with only 4M tunable parameters. Among all the models, we observed that ViT-H has the largest model size with 0.6B parameters but requires 259B GFLOPS per image and achieves only 10 frames per second (FPS) on a single A100 GPU. In contrast to our base ViT-B model only requires 32B GFLOPS per image and processes 34 FPS, resulting in a 27× improvement in computational efficiency (FPS per GFLOPS) compared with the ViT-H model. As in real-time clinical deployment, inference time is critical; thus, ViT-B was the ideal choice as it can process 34 images per second at 34 FPS, enabling faster needle tracking during interventional procedures. At the same time, the throughput in contrast to ViT-L drops to 16 FPS (only 27% of ViT-B), while ViT-H is 29% of ViT-B without any performance gains. During our experimental evaluation, the performance gains from larger backbones were a bottleneck as the computational cost of ViT-L (3.8× more GFLOPS) and ViT-H (8.1× more GFLOPS) did not improve the segmentation accuracy for the dual-target needle and tumor segmentation tasks. Therefore, we selected ViT-B as our default backbone over a large model as it balances model performance across both classes segmentation accuracy, inference speed, and computational efficiency (1.06 FPS/GFLOPS) for real-world clinical systems.

**Table 2:** Needle localization results across four evaluation datasets: test set, unseen datasets, and unseen *in vivo* human clinical data.

Method	Test Set				Unseen (18G)				Unseen (23G)				Unseen Human clinical Data			
	MHD	TE	NLSR	NLR	MHD	TE	NLSR	NLR	MHD	TE	NLSR	NLR	MHD	TE	NLSR	NLR
	(mm)	(mm)	(%)	(%)	(mm)	(mm)	(%)	(%)	(mm)	(mm)	(%)	(%)	(mm)	(mm)	(%)	(%)
UIU-Net [19]	1.90	3.57	100	92	0.79	3.21	99	95	2.20	2.74	100	95	3.73	2.03	100	83
ASCENT+ [24]	1.51	1.09	100	94	1.32	0.80	100	98	1.69	2.39	100	94	0.47	1.03	100	86
SAM [30]	5.71	11.96	0	0	4.81	11.89	0	0	6.34	14.94	0	0	8.05	5.80	0	0
MedSAM [34]	10.25	5.23	44	70	5.13	3.47	96	71	4.41	14.69	11	73	3.75	12.23	88	91

MedSAM (fine-tuned)	1.48	1.22	99	86	1.42	1.30	99	82	0.51	2.17	75	81	0.38	3.77	100	94

**USPA-SAM (ours)**	**0.34**	**0.83**	**100**	**92**	**0.25**	**0.71**	**100**	**99**	**0.27**	**1.65**	**100**	**98**	**0.64**	**2.46**	**100**	**96**

**Fig. 3:**
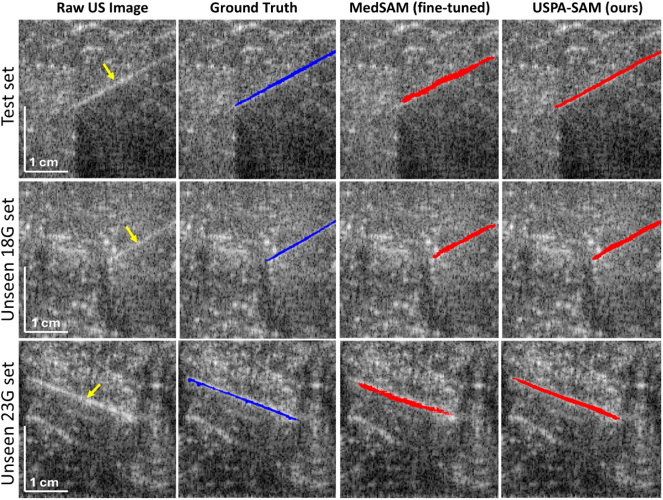
Needle localization results on test set, unseen 18G, and 23G *ex vivo* dataset. All metrics are reported as mean values.

### 3.3 Performance evaluation

The comprehensive performance comparison of our primary USPA-SAM model for needle tracking on different datasets is presented in Table 2 from our standardized adaptation protocol. For ablation analysis, we also fine-tuned the MedSAM, a variant of the FM for medical imaging using our parameter-efficient adaptation strategy for comparative analysis along with other baselines, such as UIU-Net, ASCENT+, SAM, and MedSAM, as reported in Table 2. For the fine-tuned MedSAM, we used their pre-trained model that trained on large-scale medical images. To train this model, the image and prompt encoder components were frozen, while the mask decoder was only a trainable parameter using AdamW optimizer (lr $=1\times 10^{-5}$, weight decay $=1\times 10^{-4}$) and the same unified loss function as described in the method section.

The table presents the performance and generalization capability of USPA-SAM across four different datasets (test sets, unseen 18G, 23G *ex vivo* needles dataset, and unseen clinical *in vivo* human biopsy data) to validate the robustness of the proposed model. On primary test set, USPA-SAM achieves state-of-the-art performance across all metrics by attaining a lower MHD of 0.34 mm. This represents a 17× improvement over the baseline SAM model (5.71 mm) and a 30× improvement over the MedSAM model (10.25 mm), showing the effectiveness of our parameter-efficient fine-tuning strategy. Additionally, we achieved a lower TE of 0.83 mm than all baseline methods. USPA-SAM also achieved a 100% NLSR suggesting that our model detected the needles thin tip line in every single frame. At the same time, the 92% NLR indicates that USPA-SAM can accurately detect and locate the needle regions. In contrast, the original SAM model failed, and MedSAM showed poor localization accuracy. While fine-tuned MedSAM demonstrates notable improvement over the original MedSAM, but it does not match the performance of our primary USPA-SAM approach. For instance, on the test set, USPA-SAM achieves a 0.34 mm MHD by outperforming the fine-tuned MedSAM (1.48 mm) by a factor of 4.4×. This comparison demonstrates that our primary USPA-SAM model remains significant for achieving effective performance gains rather than suboptimal tuning of the MedSAM baseline.

#### 3.3.1 Unseen needle insertion data

We evaluated 18G and 23G unseen *ex vivo* needle datasets on chicken tissue to test generalizability. USPA-SAM model demonstrates exceptional performance on different needle gauges by achieving MHDs of 0.25 mm and 0.71 mm TE on 18G datasets while achieving MHDs of 0.27 mm and 1.65 mm TE on 23G datasets. Comparing it with the original SAM and MedSAM models, our model performs best by maintaining 100% NLSR in both cases while the NLR shows 99% and 98%, respectively, to estimate needle length localization. It is important to note that the thinner 23G needles are more complex to needle segment because they contain lower contrast and more imaging artifacts. However, our model maintains robust performance even under these complex conditions, achieving the lowest MHD, TE, and the highest NLR among all existing methods. Additionally, the fine-tuned MedSAM shows improvement, although it remains lower than the USPA-SAM model.

Fig. 3 displays the results for three representative experimental datasets: test set, 18G, and 23G needle data, shown in a separate row with each dataset. The first representation shows the input raw images (the needle location is highlighted with a yellow arrow) for all three experiments, followed by the PA ground truth images (marked in blue). The predicted masks are marked in red for our USPA-SAM model and fine-tuned MedSAM model in the last two columns for each representative experimental result. The resultant predicted mask output demonstrates that our trained model can efficiently locate diverse needle insertion angles. Our proposed model is less susceptible to the low-contrast artifacts (speckle noise, hazy structures) while achieving 88% Dice and 79% IoU scores.

**Fig. 4:**
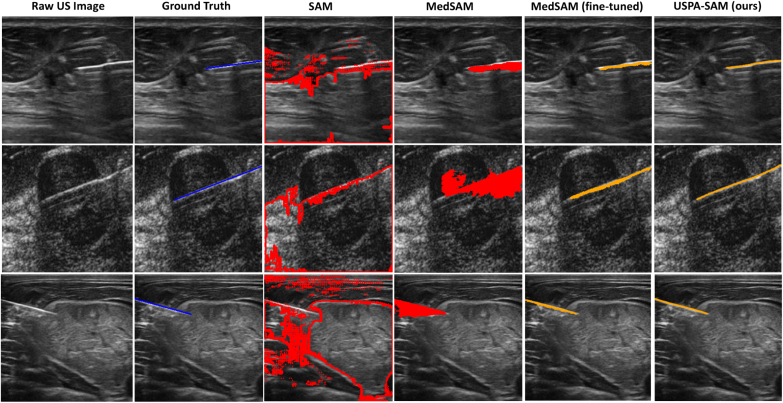
Needle localization results on unseen *in vivo* clinical human biopsy dataset. USPA-SAM (ours) and fine-tuned MedSAM is highlighted in orange, while baseline SAM and MedSAM is highlighted in red.

#### 3.3.2 Unseen clinical human dataset

We evaluated the proposed method on *in vivo* clinical human dataset (unseen) to validate the robust generalizability of US-guided needle tracking. As shown in Table 2, USPA-SAM achieved an MHD of 0.64 mm and a TE of 2.46 mm, maintaining a 100% NLSR and a 96% NLR. The fine-tuned MedSAM variant performance shows that MHD 0.38 mm is lower than all other baseline methods on this clinical dataset, although it is compromised with higher TE 3.77 mm. This implies that the predicted results are less accurate in terms of absolute needle tip position, which is a critical metric for clinical targeting. However, in terms of overall segmentation accuracy across all datasets and metrics, USPA-SAM exhibits more balanced and robust generalization.

Fig. 4 displays a qualitative comparison of our proposed model with existing baseline methods. The resultant evaluation demonstrates that our models consistently exhibit performance by eliminating the false positives prevalent in low-contrast US images. The original SAM-based model (marked red) struggles with segmenting pixels outside of the thin needle region and lacks domain-specific constraints for US imaging systems. While USPA-SAM shows the predicted mask of needle lines more accurately than fine-tuned MedSAM (both marked in orange) closely matches the PA ground truth (marked in blue) images across varying imaging conditions. These overall results indicate that our parameter-efficient fine-tuning enables significant domain adaptation, allowing the model to generalize with substantial performance gains.

**Table 3:** Tumor segmentation performance on BUSI and unseen BUS-UCLM datasets.

Dataset	Method	Count	Dice	IoU	Precision	Recall	Pixel Acc.
BUSI	U-Net [19]	780	0.7267 ± 0.3533	0.5586 ± 0.3032	0.5840 ± 0.4526	0.6028 ± 0.3627	0.9409 ± 0.0828
	SAM [30]	780	0.1167 ± 0.2245	0.0825 ± 0.1735	0.2361 ± 0.3672	0.1650 ± 0.2907	0.7655 ± 0.2333
	MedSAM [34]	780	0.9136 ± 0.0447	0.8439 ± 0.0706	0.9402 ± 0.0562	0.8921 ± 0.0603	0.9769 ± 0.0206
	**BT-SAM (ours)**	780	**0.9358 ± 0.1149**	**0.8924 ± 0.1252**	**0.9454 ± 0.1156**	**0.9286 ± 0.1214**	**0.9914 ± 0.0077**

BUS-UCLM	U-Net [19]	683	0.7530 ± 0.2086	0.5730 ± 0.1685	0.6282 ± 0.3164	0.6401 ± 0.1879	0.9804 ± 0.0431
	SAM [30]	683	0.1315 ± 0.3226	0.1250 ± 0.3175	0.1849 ± 0.3835	0.1368 ± 0.3318	0.7293 ± 0.1655
	MedSAM [34]	683	0.5034 ± 0.3082	0.4363 ± 0.2657	0.5295 ± 0.3519	0.4797 ± 0.3341	0.8630 ± 0.0551
	**BT-SAM (ours)**	683	**0.9629 ± 0.0957**	**0.9403 ± 0.1320**	**0.9719 ± 0.0858**	**0.9595 ± 0.1105**	**0.9962 ± 0.0120**

**Fig. 5:**
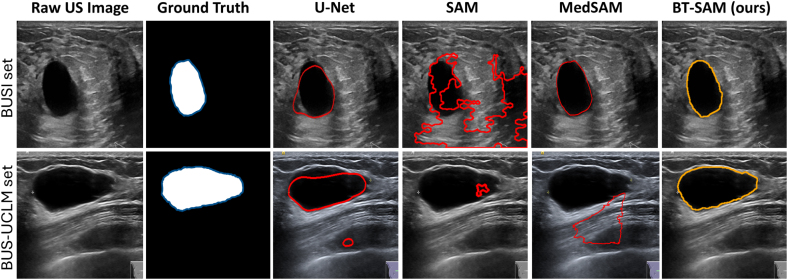
Tumor segmentation results on BUSI and unseen BUS-UCLM dataset. Our BT-SAM predicted mask is highlighted in orange, while baseline methods U-Net, SAM, and MedSAM prediction masks is highlighted in red. The ground truth masks are shown in blue overlay.

#### 3.3.3 Tumor segmentation in ultrasound images

The quantitative segmentation performance of the US breast tumor is presented in Table 3, where we employed the BUSI dataset to evaluate our proposed BT-SAM model for tumor segmentation tasks, which outperforms all baseline methods. We used a total of 780 BUSI image dataset samples, comprising normal (133), benign (437), and malignant (210) target classes. Our fine-tuned BT-SAM model achieved a Dice score of 93.6%, an IoU of 89.2%, a precision of 94.5%, a recall of 92.9%, and a pixel accuracy of 99.1% compared to baseline supervised models. In comparison with MedSAM, the best model demonstrates impressive results (91.4% Dice and 84.4% IoU) across all metrics, surpassing the original SAM model. In comparison, we generalized our model using the unseen BUS-UCLM dataset (not used during training and fully annotation-free), which shows a significant performance improvement of 96.3% in Dice score, 94.0% in IoU, 97.2% in precision, 96.0% in recall, and 99.6% in pixel accuracy, with a moderate computational cost. This demonstrates the robust and reliable performance of BT-SAM for breast tumor image segmentation in ultrasound imaging, with minimal false positives/negatives. Additionally, the quantitative results of BT-SAM demonstrate model stability in terms of zero-shot segmentation accuracy, with lower false positives due to the low standard deviations.

Fig. 5 illustrates that our BT-SAM model shows accurate tumor segmentation and achieves higher Dice and IoU scores compared to the baseline methods. While U-Net and MedSAM show slightly better results on BUSI data, MedSAM produces poorer performance on unseen data compared to traditional U-Net methods. However, the original SAM model struggles to outline the edges accurately due to the domain gap.

**Fig. 6:**
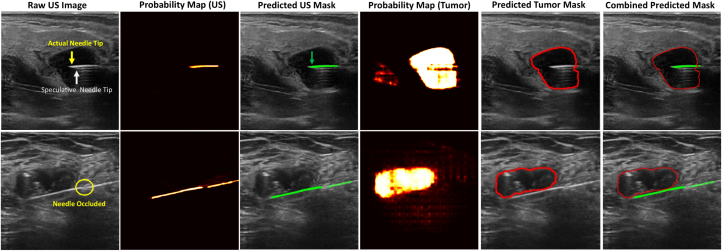
Dual-target segmentation of US images of breast cyst (tumor) and biopsy needle with its corresponding probability map features. Predicted needle mask (green) and tumor mask (red).

#### 3.3.4 Dual-target class segmentation

The novel approach of our dual-target segmentation results is illustrated in Fig. 6. During the training phase, we provided the PA ground truth bounding box as a prompt, allowing the mask decoder to learn the different target classes of US images. This enables the decoder to filter the class of distinct visual features in the image embedding. The loss function is computed simultaneously for both segmentation classes to compare with the predicted output masks. This helps the model to learn specific output tokens for each class from a single forward pass using distinct masks for the needle and the tumor.

We evaluated and validated our unified dual-target framework on a publicly available small set of clinical human biopsy datasets unseen during training. During the inference phase, our model weights were applied to segment both needle and tumor simultaneously on a raw US image to filter each target class to generate predicted masks, as shown in Fig. 6. The figure demonstrates the localization performance on US images using our specialized model. It shows that FM-Adapt utilizes unlabeled US images as input to query in the model during the inference stage to predict target masks for US needles (green). In contrast, the original raw images contain challenging imaging artifacts [55], including a speculative needle tip (white arrow), compared with the actual needle tip (yellow arrow), and occluded needle regions (circled in yellow). Consecutively, the tumor segmentation model effectively learns to delineate the tumor boundaries (red) and heterogeneous textures of breast lesions. The final post-processing stage of the dual-target class region combines the predicted masks of the exact needle (green) and tumor (red) to integrate a complex predicted mask into a single final output. This process projects the probability maps for both needle and tumor images into a latent anatomical space. This dual-specialization approach leverages our unified adaptation FM to segment US images accurately across any reference target image class.

## 4 Discussion

The FM-Adapt framework demonstrates that parameter-efficient fine-tuning adaptation of the SAM-based FM can overcome key limitations for simultaneous PA-supervised US-guided needle tracking and breast tumor segmentation. Our method addresses one of the drawbacks of current approaches, including reduction of computational complexity and multi-modal generalization, multi-target segmentation, while achieving good performance. Our parameter-efficient approach involves training only the mask decoder, which contains 4M parameters, while freezing the pre-trained image and prompt encoders. This reduces training time to less than 30 min on a single GPU, and decreases compute requirements by 95%, resulting in an inference speed of 34 FPS without sacrificing accuracy. Our results show USPA-SAM achieved a MHD of 0.34 mm on the test set, and maintained robust performance on unseen *ex vivo* and human clinical data. At the same time, BT-SAM obtains 93.6% Dice on the BUSI dataset, and 96.3% on the unseen BUS-UCLM tumor data.

Employing current deep learning-based architectures in clinical contexts is challenging due to their extensive training time, which demands higher computational resources, reliance on laborious manual annotation, substantial hyperparameter tuning, and limited generalization on new image data. Our parameter-efficient approach mitigates these challenges and eliminates the dependency on large amounts of supervised data. This efficiency drastically reduces the computational cost for task adaptation without full re-training, and allows deployment on cloud platforms like Google Colab. Additionally, this substantial computational advantage achieved through efficient training with fewer parameters and compact inference at 32B GFLOPS per frame ensures the model is compatible with real-time clinical procedures and needs limited resources for interventional guidance. We performed a comprehensive heterogeneous datasets across *in vivo*, *ex vivo*, BUSI, BUS-UCLM, and clinical human biopsy data to demonstrate clinical readiness and model generalization. We achieved robust performance on limited medical data by utilizing a pre-trained FM. We observed consistent performance across multiple US imaging acquisition systems (including Siemens, Fujifilm, and GE), suggesting that our model learns hardware-invariant features for precise segmentation tasks. This cross-system consistency demonstrates the robustness of our approach and effective generalization under different imaging conditions. The morphological analysis of our segmentation results for breast anatomical regions is more closely aligned with human expert-annotated labels in terms of shape, length, edges, and contours of US images.

Although the evaluation shows significant improvement, it also poses some limitations, specifically in terms of other clinical data classes and sensitivity to input prompts. This study does not make any systematic comparison of decoder-only fine-tuning against other parameter-efficient fine-tuning (PEFT) methods like LoRA [56], and quantitative analysis of PA-derived supervision against manual US annotation, leaving a scope for future work for comparative evaluation. As previously stated, the proposed approach performs well on the tested *in vivo* and *ex vivo* data from specific imaging acquisition systems. However, it is essential to consider further validation for more complex imaging conditions, including data from different imaging systems, images containing multiple or parallel needles, and other anatomical structures or modalities. Additionally, the bounding-box prompt may affect results due to some extreme cases of imaging conditions or user input quality, which can hinder optimal performance for each task. Hence, automated prompt generation could mitigate this sensitivity, whereas inaccurate prompts could compromise output results.

While this work presents a unique dual-target adaptation for segmentation tasks, several promising future research directions may emerge from our work. A significant advantage of this parameter-efficient design is that it enables seamless expansion to new clinical tasks, such as segmenting new anatomical structures (*e.g.,* liver, kidney). Other application could be focusing on multi-needle tracking for robotic-assisted [57], [58] treatments (such as irreversible electroporation or IRE), where parallel needle insertion is shown to have challenges in maintaining precise spacing, tip localization, and depth. Additionally, we will explore knowledge distillation (KD) [59], [60] between teacher and student models for model compression, pruning, and quantization techniques to facilitate deployment on edge devices (such as mobile US systems) and reduce computational bottlenecks in resource-constrained settings. Furthermore, integrating multi-modal vision language models (VLMs) such as LLaVA-Ultra [61], and LLAUS [62], which combine text-to-image and image-to-text generation, can help automate the generation of text reports with greater confidence. The multi-modal approach will benefit decision-driven insights to help clinicians during real-time interventional treatments.

## 5 Conclusion

In this work, we introduced FM-Adapt, the first parameter-efficient framework that adapts SAM for dual-target PA-supervised US-guided needle tracking and tumor segmentation. We only trained the mask decoder (4M parameters) and froze the pre-trained components to create two specialized model weights through a unified adaptation framework: USPA-SAM for needle tracking and BT-SAM for breast tumor segmentation. Our results show that both USPA-SAM and BT-SAM achieved improvements in needle and tumor segmentation tasks while maintaining computational efficiency and segmentation accuracy. USPA-SAM achieved up to 3–17× improvement in spatial accuracy for needle tracking with a MHD of 0.34 mm, a TE of 0.83 mm, and a 100% localization success rate. On BUSI and BUS-UCLM, BT-SAM achieved a Dice score of 93.6% and 96.3%, along with IoU scores of 89.2% and 94.0%, outperforming existing supervised methods. Both models achieved an inference speed of 34 FPS on standard hardware, ensuring compatibility with real-time clinical procedures. The FM-Adapt framework demonstrates significant potential by offering an optimal balance between performance and efficiency for PA-supervised procedures.

## CRediT authorship contribution statement

**Jahid Hasan:** Writing – review & editing, Writing – original draft, Visualization, Software, Methodology, Investigation, Data curation, Conceptualization. **Praveenbalaji Rajendran:** Writing – review & editing, Supervision, Methodology. **Ying Cai:** Writing – review & editing, Supervision, Project administration. **Manojit Pramanik:** Writing – review & editing, Supervision, Project administration, Methodology, Investigation, Conceptualization.

## Declaration of competing interest

The authors declare that they have no known competing financial interests or personal relationships that could have appeared to influence the work reported in this paper.

## Data Availability

The source code is available on GitHub: https://github.com/DeeplearningBILAB/FM-Adapt-foundation-model-PA-US-segmentationand the datasets can be downloaded from Open Science Framework (OSF): https://osf.io/pu86k/overview.
